# Pharmacological, nutraceutical, and nutritional properties of flaxseed (*Linum usitatissimum*): An insight into its functionality and disease mitigation

**DOI:** 10.1002/fsn3.3662

**Published:** 2023-10-03

**Authors:** Sana Noreen, Tabussam Tufail, Huma Bader Ul Ain, Chinaza Godswill Awuchi

**Affiliations:** ^1^ University Institute of Diet and Nutritional Sciences, The University of Lahore Lahore Pakistan; ^2^ School of Food and Biological Engineering, Jiangsu University Zhenjiang China; ^3^ School of Natural and Applied Sciences Kampala International University Kampala Uganda

**Keywords:** dietary measures, flaxseed, functional foods, health benefits

## Abstract

Flaxseed (*Linum usitatissimum* L.) is derived from the flax plant, an annual herb. The primary relevance of flaxseed is in the human nutrition sector, where it is emerging as a significant functional food component due to its high level of active chemicals, which have been linked to health benefits. Flaxseed may be consumed in numerous forms, including milled, oil, and bakery items. The phytochemicals that are present in flaxseed have greatly drawn interest as bioactive molecules beneficial for health. It is naturally enriched with alpha‐linolenic acid, omega‐3 fatty acid, lignin, secoisolariciresinol diglucoside, and fiber which are physiologically active in the protection of some chronic illnesses such as cancer, diabetes, cardiovascular disease, and cerebrovascular stroke. Furthermore, the benefits of flaxseed eating have been demonstrated in the animal nutrition industry, resulting in healthier food from animal origin. In reality, the fatty acid profile of meat and fat in swine and poultry is directly impacted by the source of fat in the diet. Feeding omega‐3‐enriched diets with flaxseed will improve the omega‐3 content in eggs and meat, enriching the products. The current study focuses on the latest evidence on the chemical makeup of flaxseed and its positive benefits.

## Graphical abstract

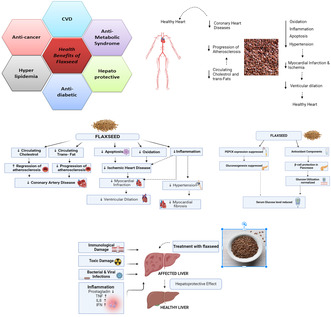

## 1 INTRODUCTION

Dietary flaxseed (*Linum Usitatissimum*) has a substantial and expanding study literature supporting its use in a wide range of health issues. In the late twentieth century, little was understood about the health‐related advantages of flaxseed and how to ingest flaxseed to get any health benefits. The amount of research on the benefits of eating flaxseed has expanded considerably (Brown et al., 2018; Noreen, Rehman, et al., 2023; Noreen, Tufail, Badar Ul Ain, Awuchi, 2023; Noreen, Tufail, Badar Ul Ain, Ali, et al., 2023; Noreen, Tufail, Tufail, et al., 2023). The bioactive found in flaxseed provide these health benefits in many circumstances, and the forms of flaxseed that are necessary to supply these bioactive to the body. Flaxseed from the *Linaceae* family is a type of herb that has been observed to have significant beneficial effects which mainly include the reduction of blood lipids (Prasad, 2019), anti‐diabetic characteristics, hepatoprotective effects, and prevention of many cardiovascular diseases (CVDs) and cancer. The amount of lin plant foods is highest in flaxseeds, which is about 800 times greater than in other plant foods consumed (Patil, 2017). Scientists evaluated a water‐soluble flaxseed extract and identified excess proteins, water‐soluble carbohydrates, and phenolic chemicals, particularly secoisolariciresinol diglucoside (SDG), ferulic acid, pinoresinol, lariciresinol, and matairesinol (Waszkowiak & Barthet, 2015; Noreen, Rehman, et al., 2023; Noreen, Tufail, Badar Ul Ain, Awuchi, 2023; Noreen, Tufail, Badar Ul Ain, Ali, et al., 2023; Noreen, Tufail, Tufail, et al., 2023). During the ingestion of flaxseeds, SDG metabolized and converted into two mammalian lignans, Enterodiol (END) and Enterolactone (ENL) with the help of gut microbiota (Taibi et al., 2021). In the top region of the colon, gastrointestinal bacteria release Secoisolariciresinol (SECO), the non‐sugar component of SDG, which is hydroxylated and demethylated to produce the mammalian lignan END, which is oxidized to produce ENL. ENL and END generated are well absorbed in the bloodstream or undergo conjugation by enterocytes to inactive compounds excreted in the gut (Kajla et al., 2015).

In this review, we closely studied the recent and past developments in the nutritional and pharmacological properties of flaxseed and its suitability in the formulation of functional foods. Our study demonstrates (a) the extensive applications/uses of flaxseed and its components, (b) the existing and emerging potential benefits of flaxseed in human health, (b) the application of flaxseed and its derivatives in treating/managing diseases such as acute myeloid leukemia (AML), CVDs, hyperlipidemia, diabetes mellitus, metabolic syndrome, etc., and (d) the major components of flaxseed. Where necessary, we blended them with their underlying action mechanisms. The hepatoprotective effects of flaxseed are also systematically covered. The composition of flaxseed includes 20% protein, 41% fat, and 28% fiber. The protein present in flaxseed is similar to that of soy protein. Lignan is another important component found in flaxseed (Suri et al., 2020). The major portion of flaxseed is composed of α‐Linolenic acid (ALA). It has been observed that ALA reduces the risk of cardiac diseases through a reduction in triglyceride and serum cholesterol amounts present in the body (Goyal et al., 2018). Moreover, ALA has beneficial effects on inflammation and lipid profiles. Several animal researchers have proclaimed that the effect of the oil of flaxseed on the decrease of phospholipids, serum cholesterol levels, and levels of triglycerides is mainly due to its major portion of ALA. The interaction of major compounds present in flaxseed including ALA, fiber, and lignin complex is mainly responsible for the hypocholesterolemic effects of flaxseed (Tufail et al., 2023; Xie et al., 2019). Additionally, flaxseed is an excellent source of antioxidants and phytosterols. Phytosterols play a crucial role in the reduction of low‐density lipoprotein (LDL) levels by inhibiting the absorption of cholesterol and possessing anti‐inflammatory and anti‐oxidant properties (Moreau et al., 2018). It has been estimated that plasma LDL levels can be decreased by 9%–14% by consumption of 1.5–2.0 g of plant sterols/day (Vilahur et al., 2019).

## 2 USES AND IMPORTANT APPLICATIONS OF FLAXSEED

Flaxseed's primary bioactive constituents include ALA, lignans, and fiber. Whole flaxseed, ground flaxseed, flaxseed oil, and partly defatted flaxseed meal are the four most prevalent forms of flaxseed accessible for human consumption. As a functional food ingredient, flax or flaxseed oil has been incorporated into baked foods, juices, milk and dairy products, muffins, dry pasta products, macaroni, and meat products. Baked products are the most common foods containing flaxseed. Baking at temperatures as high as 178°C for 2 h, for example, had no effect on the composition or concentration of ALA in a cooked muffin (Chen et al., 1994; Noreen, Rehman, et al., 2023; Noreen, Tufail, Badar Ul Ain, Awuchi, 2023; Noreen, Tufail, Badar Ul Ain, Ali, et al., 2023; Noreen, Tufail, Tufail, et al., 2023). The addition of flavoring to baked goods also allows for the masking of any less‐than‐ideal flavor character caused by flaxseed bitterness or slight rancidity. The processing of the seed, storage temperature, and length, as well as the shape of flaxseed (milled flaxseed versus whole seed vs. flax oil) will all have an impact on the product's stability. By milling, grinding, or pulverizing flaxseed, the protective seed coating is destroyed, exposing the ALA and SDG to oxidation (Edel et al., 2015). This procedure, however, is essential to make these bioactive chemicals accessible. When ALA is in oil or milled form, it is more bioavailable to the body. The use of milled flaxseed in baked goods may actually preserve the ALA and SDGs from degradation. Cooler storage temperatures and shorter storage durations will also increase ALA and SDG retention, particularly in flax oil (Edel et al., 2015; Morya et al., 2022).

## 3 POTENTIAL BENEFITS OF FLAXSEED IN HEALTH

**FIGURE 1:**
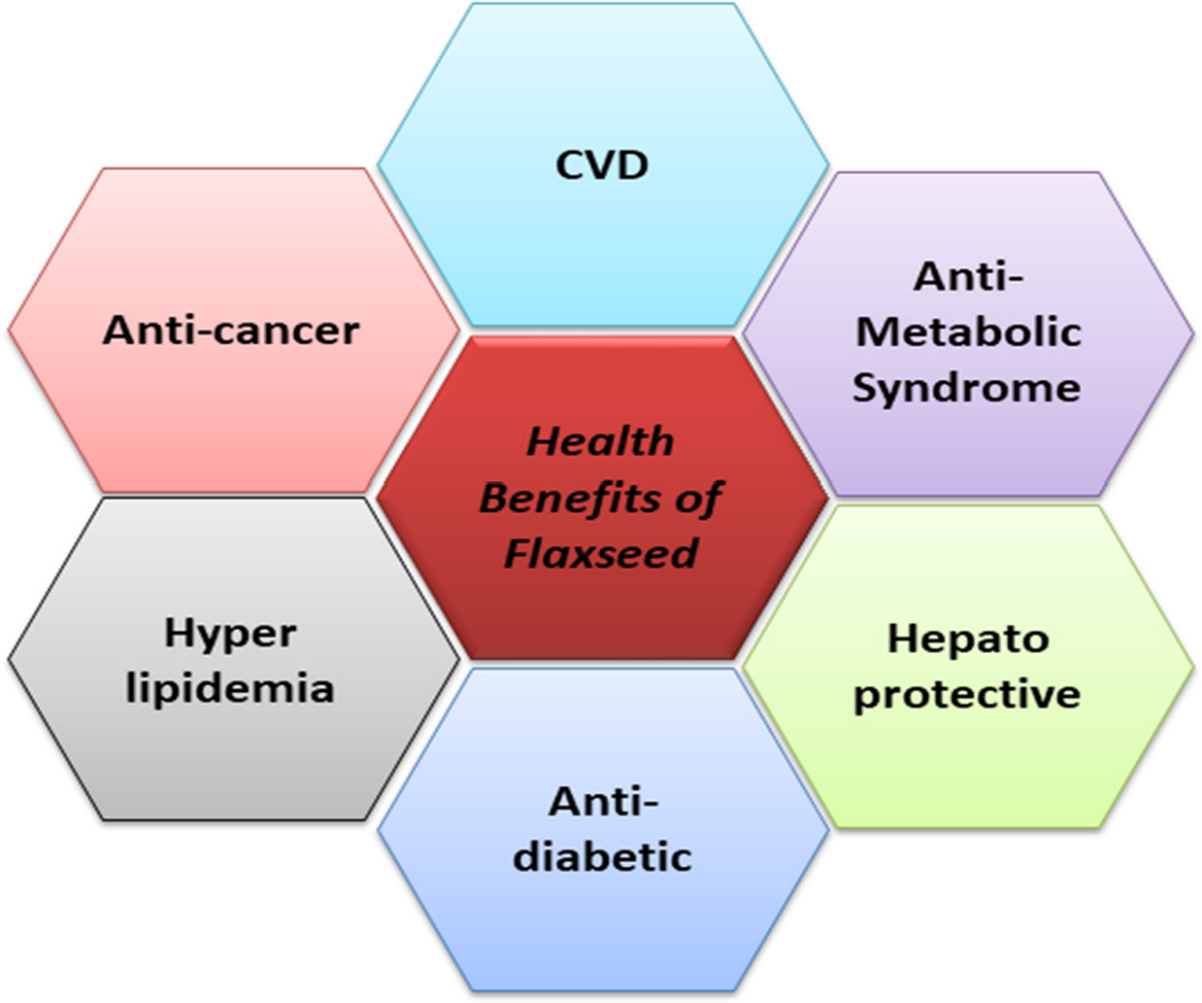
Health benefits of flaxseed.

Flaxseed is emerging as an important functional food ingredient because of its rich contents of α‐linolenic acid (ALA, omega‐3 fatty acid), lignans, and fiber. Flaxseed oil, fibers, and flax lignans have potential health benefits such as in reduction of CVD, atherosclerosis, diabetes, cancer, arthritis, osteoporosis, autoimmune and neurological disorders. Flax protein helps in the prevention and treatment of heart disease and in supporting the immune system. The present review focuses on the evidence of the potential health benefits of flaxseed through recent studies on humans and animals and commercial use in various food products, as seen in Figure 1, while Table 1 describes the pharmacological, therapeutic, and functional properties of flaxseed.

**TABLE 1:** Pharmacological, therapeutic, and functional properties of flaxseed.

Sr. no.	Activity	Flaxseed	Study type/cell line	Effect	Reference
1	Anti‐cancer	Flaxseed derivatives	In vivo and In vitro	Possesses therapeutically beneficial anti‐cancerous effects	Eriksen et al. (2017), Mali et al. (2019)
		Flaxseed derivatives	Two AML cell lines, namely KG‐1 and Monomac‐1, in vitro	Promising anti‐cancerous effect	Tannous et al. (2020)
		Flaxseeds lignan	Healthy B‐lymphoblasts of human beings	Results validated their selectivity of cancerous cells targeting leaving the cells that are normal	Mali et al. (2017)
2.	Hyperlipidemia	Roasted flaxseed supplementation incorporated into chapattis	Efficacy of flaxseed supplementation on lipid profile of hypercholesterolemic patients	Decreased total cholesterol (TC), LDL‐cholesterol levels, triglyceride, (TG) and increased HDL cholesterol levels	Prasad et al. (2020)
		Flaxseed supplementation	Hyperlipidemic patients	Improvement in total cholesterol, triglyceride, and (LDL‐C) levels along with prevention as well as delayed progression of heart diseases	Hadi et al. (2020)
		Flaxseed	Severe hyperlipidemic patients	Flaxseed supplementation was tolerated well by the patients and led to exceptional and consistent decreased levels of LDL and total cholesterol	Kanikowska et al. (2020)
		Flaxseed powder (30 g for 40 days)	Lipid profile of 70 hyperlipidemic patients	Reduction in weight as well as BMI of subjects. Significant decrease in total cholesterol and low‐density lipoprotein levels was observed	Torkan et al. (2015)
3.	Anti‐diabetic	Ground flaxseed (0, 13, or 26 g consumed for 12 weeks)	25 overweight or obese women and men in postmenopausal stage and pre‐diabetic	The group that was given flaxseed dosage of 13 g/day experienced decreased glucose level as compared to the other two groups	Hashemzadeh et al. (2017)
4.		Grounded flaxseed and flaxseed oil (10% grounded flaxseed and 4% flaxseed oil)	Streptozotocin (STZ)‐induced diabetic rats	The result concluded that the diabetic rats who received 10% grounded flaxseed were more prone to improve within 8 weeks from diabetes that was induced	Prasad and Dhar (2016)
	Hepatoprotective	Flaxseed, sesame seeds and their oils	NAFLD in 54 adult male Albino rats (Sprague Dawley strain)	The result proved that significant decrease in body weight gain, organ weight, peritoneal fat pad PFP/body weight PFP, leptin, glucose, lipid profile including (cholesterol, triglycerides, LDL‐c, and VLDL‐c), liver enzymes including AST, ALT, and ALP, while these treatments induced significant increase in feed intake, HDL‐c, and antioxidant enzymes(GSH‐GPx, SOD, and CAT)	El‐Aziz et al. (2020)
5.	Anti‐ Metabolic Syndrome	Flaxseed oil (FO) and sunflower seed oil (SO)	60 volunteers aged 30–60 years were diagnosed with MetSyn	Significant reductions in total cholesterol, low‐density lipoprotein cholesterol, and triglyceride levels were seen. Effective in amelioration of some symptoms of MetSyn and reduce BP levels and lipid peroxidation	Akrami et al. (2018)
		Flaxseed oil	D‐galatosamine‐sensitized albino rats	The results obtained were that LPS/DGalN vitally enhanced serum liver functions, MDA, TNF‐α, IL‐1α, urinary 8‐OhdG concomitant with a decrease in liver GSH and SOD than the control group while, these parameters were reduced by intake of flaxseed oil	Hussein et al. (2016)
		Flaxseed oil and sunflower oil	68 NAFLD patients	Reduced fatty liver grade Decreased AST and ALT serum levels. Reduced levels of glucose in the blood and fat mass of the group given flaxseed oil and the mass of the muscles of the batch given oil made from sunflowers were observed	Rezaei et al. (2020)

Abbreviations: ALP, alkaline phosphatase; ALT, alanine aminotransferase; AML, acute myeloid leukemia; AST, aspartate aminotransferase; HDL, high‐density lipoprotein; LDL, low‐density lipoprotein; LPS, lipopolysaccharide; NAFLD, non‐alcoholic fatty liver disease; VLDL, very low density lipoprotein.

## 4 FLAXSEED DERIVATIVES AND AML

Flaxseed lignans are potentially beneficial for cancer therapy as preceding studies have been observed to portray reported in vivo and in vitro anti‐cancerous effects. Although flaxseed lignans have portrayed remarkable potentiality in the prevention and treatment of a number of different types of cancers. AML, acute leukemia in adults (De Kouchkovsky & Abdul‐Hay, 2016), emerges by clonal expansion of myeloid progenitors in the bone marrow or in peripheral blood. Prior researchers have analyzed that the collection of genetic variations with increasing age is a contributing factor for this disorder which leads to the proof that the severity of the disease enhances with age (Mueed et al., 2022; Saultz & Garzon, 2016). Flaxseeds have been used anciently as an antitumor treatment, they possess therapeutically beneficial anti‐cancerous effects, both in vivo (Eriksen et al., 2017) and in vitro (Mali et al., 2019). According to research, flaxseed derivatives were analyzed to verify the effect on two AML cell lines, namely KG‐1 and Monomac‐1 cells, in vitro, and promising results were obtained (Tannous et al., 2020). A number of previously conducted researches have shown that ENL is the most vital lignan derivative of SDG, due to its ability to treat breast cancer (Liu et al., 2017). Also, ENL is greatly bioavailable in blood on formation in the gut, knowing that these lignans are produced, metabolized, absorbed, and excreted variably among individuals, because of the varied microflora of individuals and also the unique hepatoportal system (Eriksen et al., 2017). As well as, the result of ENL on the cells lines activity observed in prostate cancer and those observed in breast cancer cells like MDA‐MB‐231, being aware of the fact that lignans are incredibly active against the cells of breast cancer (Mali et al., 2017).

Flaxseeds’ lignan activity was also studied on healthy B‐lymphoblasts of human beings, this investigation led to results validating their selectivity of cancerous cells targeting leaving the cells that are normal. This characteristic is similar to studies carried out previously where ENL was observed to portray inhibition of proliferation of a prostate cancer cell line LNCaP along with modified prostate cell line WPE‐1 with limited effects on the normal prostate cells (Demark‐Wahnefried et al., 2008). ENL leads to cell death, that is, apoptosis. It was proved by advancement in cellular fragmentation, the turnover of the phosphatidylserine moiety toward the outer side of the cell membrane explaining the enhanced staining. A group of apoptosis‐promoting proteins (bax, bak, bcl‐xS) or apoptosis‐inhibiting proteins (bcl‐2, mcl‐1, bcl‐xL) was formed bcl‐2 family proteins which played a vital part. These proteins regulate the permeability of the mitochondrial outer membrane, as well as the irreversible release of intermembrane space proteins, followed by caspase activation and apoptosis (Kale et al., 2018) ENL exhibits a slight upregulation of Bax and a downregulation of Bcl‐2, thus resulting in an increase in the Bax/Bcl‐2 ratio. Moreover, Cytochrome c overexpression proved the start of apoptotic mechanisms upon enhanced ENL contact in comparison with the control (Suofu et al., 2017; Tannous et al., 2020). p‐53 portrays an apoptotic role, due to its part as a tumor suppressor gene it can be activated in both the pathways that are extrinsic and intrinsic. p‐53‐dependent apoptosis is expected to occur through the intrinsic pathway in place of the extrinsic death receptor pathway, therefore, mitochondrial intrinsic pathway is more integral. This is observed when there is a certain extent of elevation in p‐53, also other research has been reported to show p‐53‐independent apoptotic pathways (Miyake et al., 2012).

## 5 FLAXSEED DERIVATIVES AND CVDs

**FIGURE 2:**
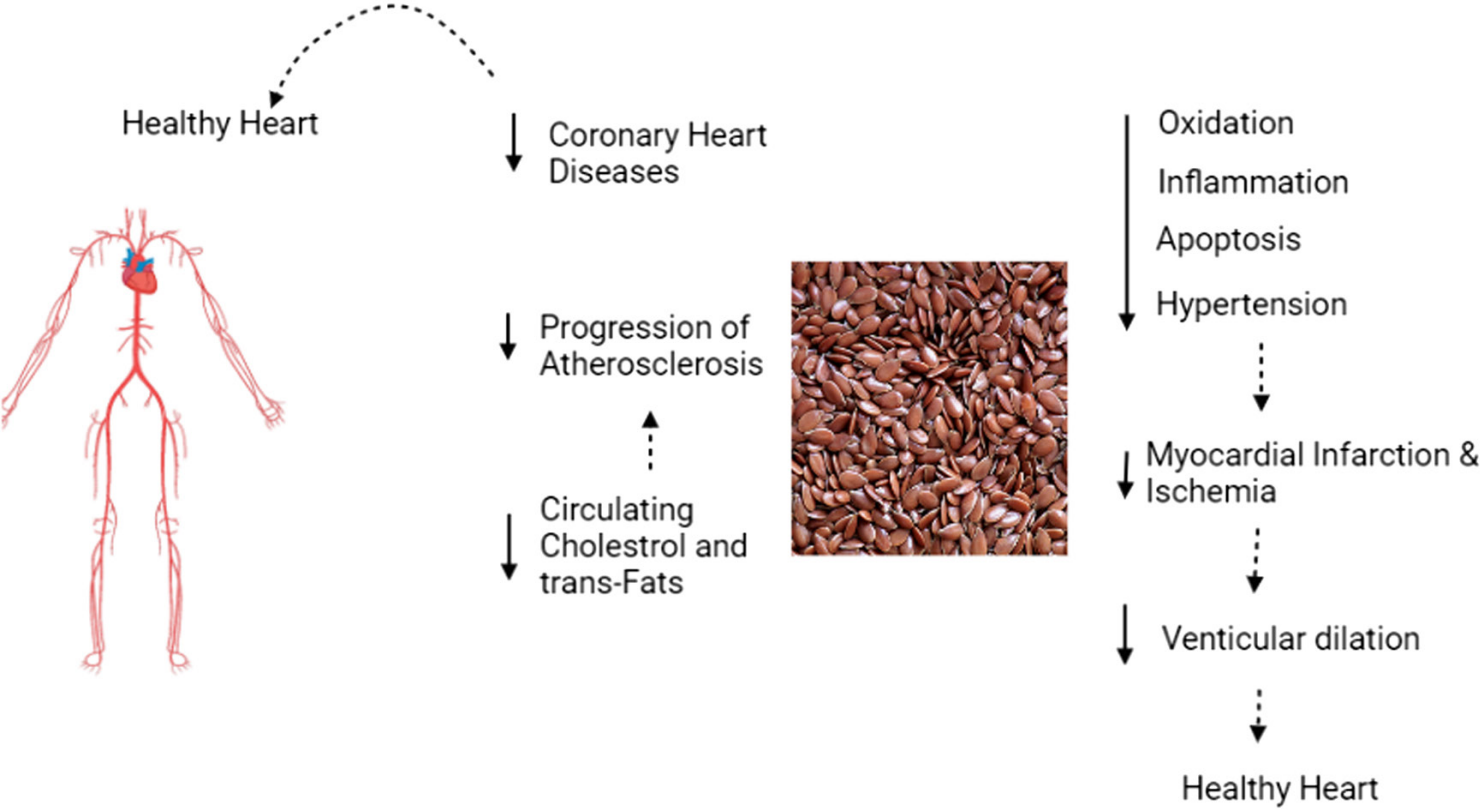
Flaxseeds improve heart health by reducing the progression of atherosclerosis, inflammation, and cholesterol level.

Nutritional management is considered an effective preventive strategy for further complications, for example, decreasing the consumption of saturated fatty acids (SFAs) can prevent the incidence of CVDs as it is effective for the management of total cholesterol levels in our body (Figure 2). It has been observed that the replacement of polyunsaturated fatty acids with saturated fatty acids can prevent CVDs (Kleber et al., 2018).

**FIGURE 3:**
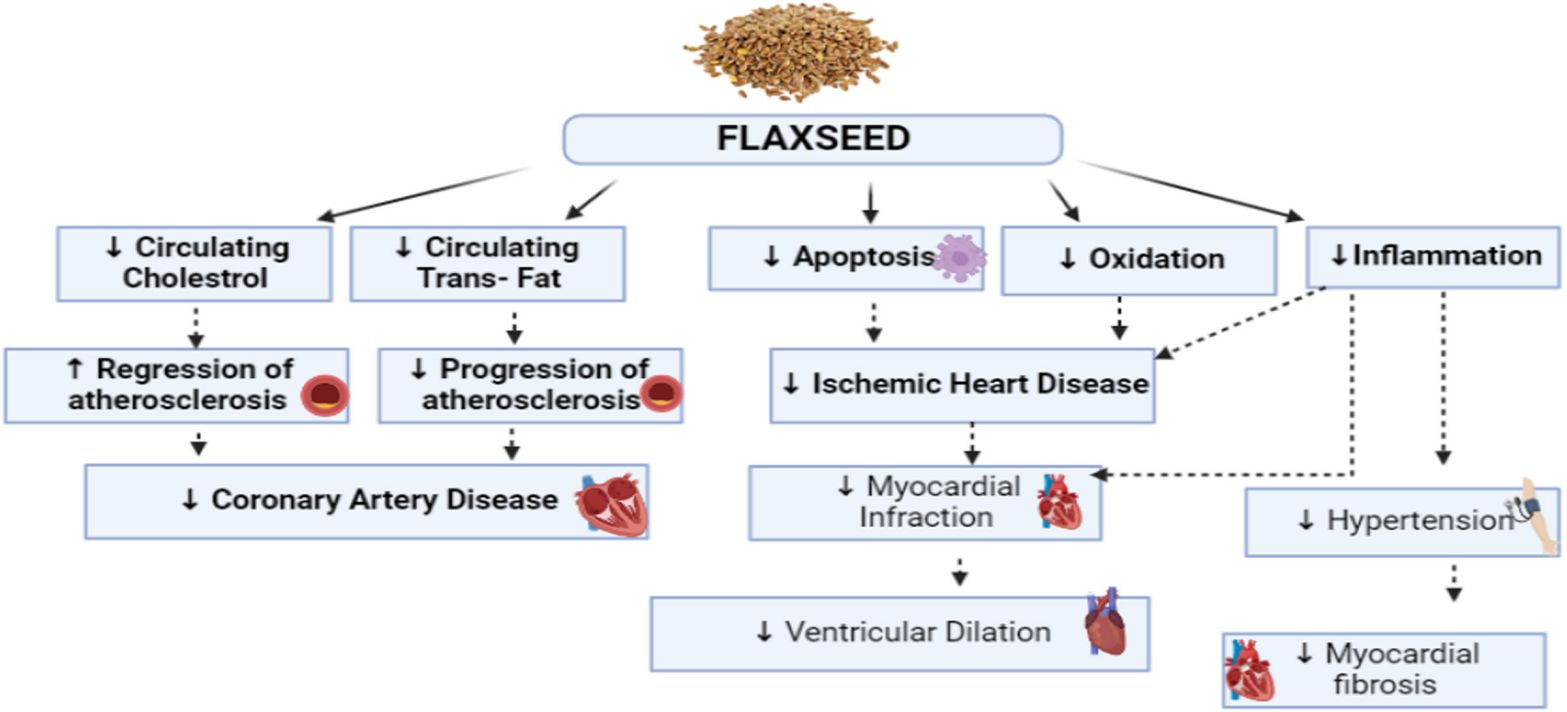
Flaxseed influences many cardiovascular diseases (CVDs) by reducing many factors that may increase the risk of CVDs.

Many epidemiological studies and researches have demonstrated that nutritional factors such as isoflavones, plant sterols, grains, and soy protein can lead to improvement in blood lipid profile. Using herbs and seeds is considered as another effective therapeutic strategy to reduce CVD risks. Dietary flaxseed possesses effective and increasing research along with vast literature that supports its use and beneficial effects for a variety of health conditions (Parikh et al., 2019). Figure 3 shows how flaxseed and its components may reduce the risk of CVDs. Many markers and factors that increase the risk of CVDs are ameliorated by flaxseed and its components (Parikh et al., 2019).

Approximately 65% of people suffering from metabolic syndrome are hypertriglyceridemic and 69% are hypercholesterolemic; however, 40% of patients with renal diseases are hyperlipidemic (Pilar et al., 2017), which clearly indicates that the incidence of high blood lipid is increasing drastically. The formation as well as the progression of atherosclerosis plaques are mainly caused by hyperlipidemia which ultimately leads to coronary heart disease. Nutritional interventions are of great importance in the prevention of cardiac disease by lowering blood lipids, as 30% of the incidence of CVD is reduced by a 10% reduction of total serum cholesterol levels (Hadi et al., 2020). Numerous studies have demonstrated that a positive lifestyle change, nutritional modifications, cessation of smoking, and increase in physical activity can prevent CVD (Pilar et al., 2017; Villarreal‐Renteria et al., 2022). The aim of this article is to review flaxseed's effects on various health conditions including hyperlipidemia, diabetes, and liver disorders.

## 6 FLAXSEED AND HYPERLIPIDEMIA

Hyperlipidemia is very common and the leading cause of increasing disease and mortality these days. It is further associated with many life‐threatening complications such as ischemic heart disease and other cardiovascular issues. It is rich in lignans and many nutrients which include soluble fiber and dietary fiber along with fatty acids that is omega 3. Omega 3 is essential for the prevention and reduction of CVD, as well as for a significant reduction in serum cholesterol and triglyceride levels. The purpose of this study was to investigate the effectiveness of flaxseed supplementation on the lipid profile of hypercholesterolemic patients. Chapattis containing roasted flaxseeds were evaluated for their flavor. Ingestion of roasted flaxseed powder for 4 weeks significantly reduced total cholesterol, LDL‐cholesterol, and triglyceride levels while increasing high‐density lipoprotein cholesterol levels in hyperlipidemic subjects (Prasad et al., 2020).

It has been reported that flaxseed supplementation improves total cholesterol, triglyceride, and low‐density lipoprotein cholesterol (LDL‐C) levels, as well as prevents and delays the progression of cardiac disease (Hadi et al., 2020). The research was conducted in order to investigate whether flaxseed supplementation could lead to an improvement in lipid profiles and inflammatory mediators in severe hyperlipidemic patients undergoing lipoprotein apheresis and resistant to lipid‐lowering conventional pharmacotherapy. The results revealed that flaxseed supplementation was tolerated well by the patients and led to exceptional and consistent decreased levels of LDL and total cholesterol. However, it had no significant effects on lipoprotein a, interleukin 6 (IL‐6), and C‐reactive protein (CRP) (Kanikowska et al., 2020; Noreen, Rehman, et al., 2023; Noreen, Tufail, Badar Ul Ain, Awuchi, 2023; Noreen, Tufail, Badar Ul Ain, Ali, et al., 2023; Noreen, Tufail, Tufail, et al., 2023). A report suggested that 15 g of ground flaxseed consumed by hyperlipidemic patients on long‐term consumption of Vitamin E (800 IU per day) significantly reduced serum cholesterol in elderly subjects enrolled in a 3‐month feeding trial. The supplement leads to a decline in levels of serum total cholesterol and LDL but high‐density lipoproteins remain unaffected in this study. However, the supplementation of flaxseed also decreased platelet aggregation stimulated by thrombin in hyperlipidemic patients (Kanikowska et al., 2020).

The effects of various fat sources on the lipid profiles of rats consuming high‐fat diets was the subject of another study. Lipid profiles of adult male Wister rats fed polyunsaturated (PUFAs) (flaxseed and trout), saturated (SFAs) (chicken skin), and monounsaturated (MUFAs) (peanuts) fatty acid sources were analyzed. The results revealed that levels of total cholesterol of rats who received a diet enriched with flaxseeds were lower (*p* < .05) than rats given other sources of fat, which concluded that dietary consumption of flaxseed is effective for the treatment of hyperlipidemia as it significantly decreased the serum total cholesterol levels of rats (Naik et al., 2018). Another previous research revealed the prospective effects of flaxseed and soy protein on hypertriglyceridemia and liver steatosis associated with diabetes and obesity. The effects of casein were compared to those of plasma and hepatic lipids in a genetic model of SHR/N‐cp rats with insulin resistance, obesity, and diabetes. In comparison to casein, flaxseed and soy protein reduced the total cholesterol levels of lean rodents by 20.3% and 26.0%, respectively. However, flaxseed‐containing diets substantially decreased cholesterol levels in obese rats compared to rats in the control group by 41%. Therefore, it could be concluded that flaxseed supplementation is a novel strategy for improving hypertriglyceridemia and obese liver (Yari et al., 2021). Clinical research was conducted to examine the effects of flaxseed powder on the lipid profiles of hyperlipidemic patients. Patients got 30 g of unprocessed flaxseed powder for 40 days. Flaxseed powder lowered weight and BMI. After supplementation with flaxseed powder, total cholesterol and LDL levels in hyperlipidemic patients decreased significantly (Torkan et al., 2015). These studies suggest that flaxseed may be an effective treatment diet for hyperlipidemia.

## 7 ANTI‐DIABETIC EFFECT OF FLAXSEED

**FIGURE 4:**
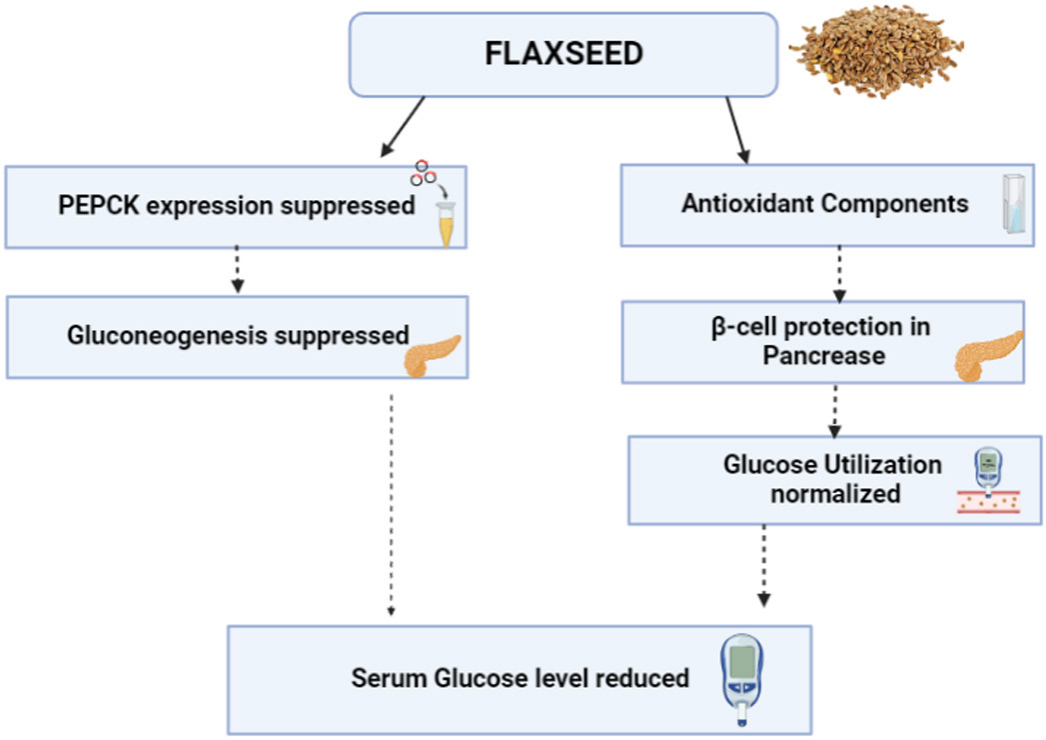
Mechanism of anti‐diabetic effect of flaxseed; part of this mechanism includes the suppression of phosphoenolpyruvate carboxykinase (PEPCK) and gluconeogenesis, the protection of beta cells in the pancreas, and normalization of glucose utilization.

Diabetes can be prevented using nutritional interventions other than medicinal therapies. This has been proved by recent studies that suggest the use of flaxseed in diet may benefit diabetics by reducing insulin resistance and it also functions as a preventive measure in decreasing the risk of developing diabetes. As flaxseeds are inherently rich in omega‐3 and omega‐6 fatty acids, they are believed to inhibit the progression of diabetes by maintaining insulin sensitivity in phospholipid membranes (Bhardwaj et al., 2015). Based on a number of epidemiological studies, clinical trials, and experiments, flaxseed intake has been observed to positively impact people suffering from diabetes and also pre‐diabetics. Several other components of flaxseed include 32%–45% of its mass as oil, 51%–55% of the oil comprised of alpha‐linolenic acid and linoleic acid about 15%–18%. A lignin abundantly found in Flaxseed is called SDG. The flaxseed content of SDG ranges from 0.6 to 1.8 g/100 g. Cinnamic acid and SDG metabolites are antioxidants and also possess a hypoglycemic effect. SDG has a hypoglycemic effect due to restrained phosphoenolpyruvate carboxykinase (PEPCK) gene expression. PEPCK is an enzyme that limits the rate of gluconeogenesis in the liver (Prasad & Dhar, 2016). The possible mechanisms of the anti‐diabetic effects of flaxseed can be seen in Figure 4. Hashemzadeh et al. (2017) conducted a study on 25 overweight or obese postmenopausal women and men who were also pre‐diabetic. The participants were given 0, 13, or 26 g of grounded flaxseed to consume for 12 weeks. According to the results, the group that received 13 g of flaxseed per day had significantly lower glucose levels than the other two categories (Hashemzadeh et al., 2017). Another study showed that moderate amounts of flaxseed consumption had favorable results in lowering blood glucose, whereas low or high dosage of flaxseed had no effect on glycemic control (Mohammadi‐Sartang et al., 2018).

## 8 HEPATOPROTECTIVE EFFECT OF FLAXSEED

**FIGURE 5:**
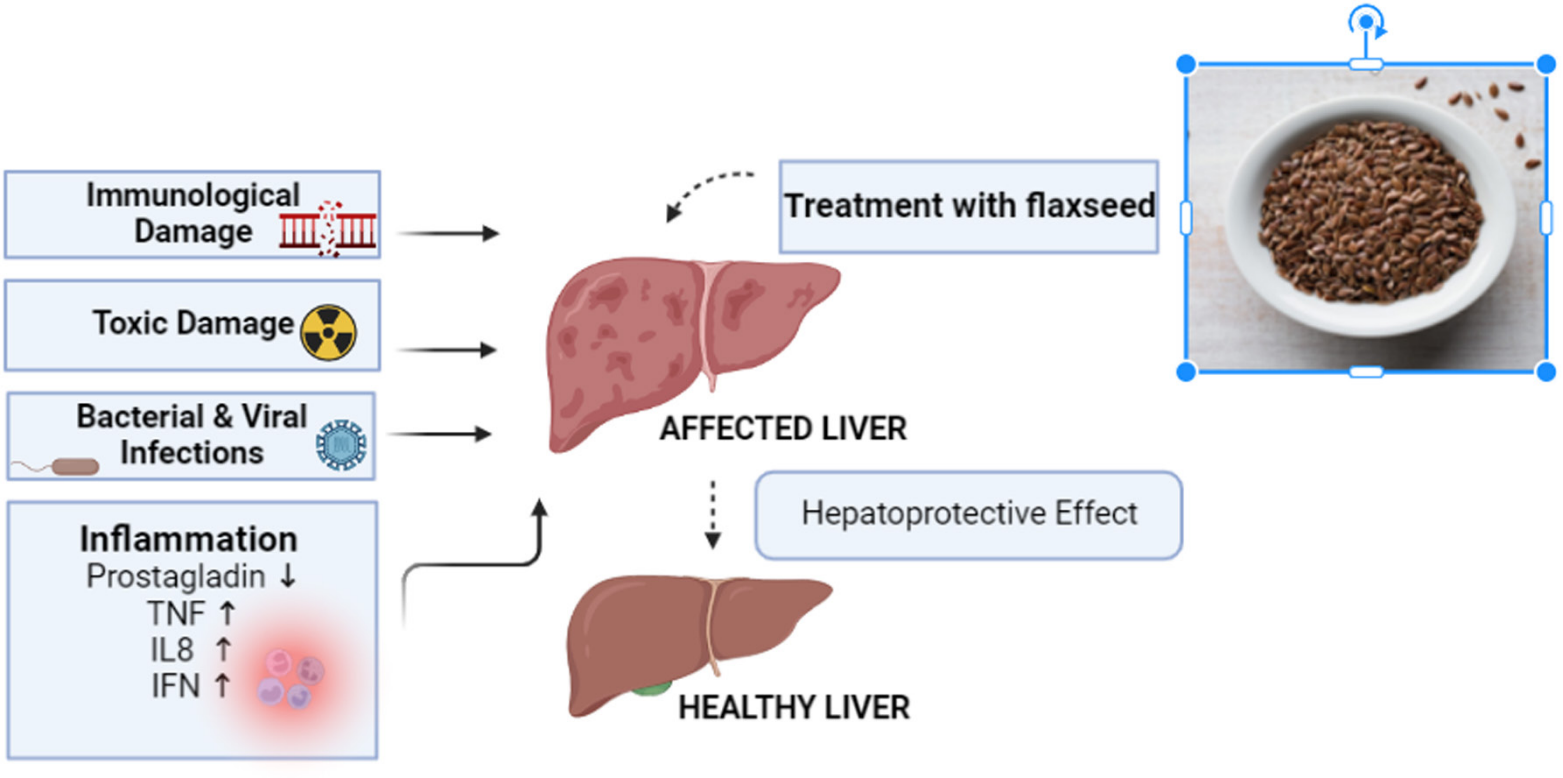
Flaxseeds show hepatoprotective effect by reducing immunological and toxic damage, infections, and inflammation.

The liver is the key player in our body system and performs a number of vital functions. Everything that we eat or drink, including medicines passes through it. It acts as a blood cleanser as well as stores glucose and controls body fuel regulation. Other functions of the liver include producing essential body proteins, hormonal balance maintenance, and regulation of blood cholesterol and vital vitamins and minerals supply. Due to its diverse functions and responsibilities, the liver is susceptible to attack by toxins, diseases, and viruses. To prevent this, nutritional modifications that provide hepatoprotective effects are preferable to supplements and medications that can cause adverse effects. Therefore, numerous studies on the hepatoprotective properties of flaxseeds have been conducted (Ahmad et al., 2017). Figure 5 illustrates the hepatoprotective effect of flaxseed.

A study was conducted on flaxseed, sesame seeds, and their oils in relation to non‐alcoholic fatty liver disease (NAFLD) in rats, using nutritional and biochemical parameters as well as determining the chemical composition and fatty acids of the two varieties of the tested seeds. Treatment of NAFLD rats with a medium‐fat diet (MFD) containing 10% flaxseed oil and sesame oil resulted in a significant decrease in body weight gain%, organ weight%, and peritoneal fat pad PFP/body. The health of rats with NAFLD was enhanced by a diet containing flaxseeds and sesame seeds (Zhang et al., 2017). Another study showed that participants received 30 g/day of flaxseeds for 12 weeks with lifestyle modification (LM) improvement in weight reduction, hepatic biomarkers, insulin resistance, hepatic fibrosis, and steatosis. In conclusion, flaxseed supplementation combined with LM is more effective than LM alone for the management of NAFLD (Yari et al., 2016).

## 9 FLAXSEED DERIVATE WORK ON METABOLIC SYNDROME

Metabolic syndrome (MetSyn) increases the risk of type II diabetes and morbidity and mortality due to CVDs. Flaxseed oil (FO), as a functional food, is one of the major vegetal sources of essential omega‐3 fatty acids. This randomized, controlled interventional trial was conducted on 60 MetSyn‐diagnosed volunteers aged 30–60 in Shiraz, Iran. Using block randomization, participants who met the inclusion criteria were randomly assigned to the SO (*n* = 30, receiving 25 mL/d SO) and FO (*n* = 30, receiving 25 mL/d FO) groups. Dietary requirements were identical for all participants. At baseline and on the 7th week, blood pressure (BP), serum lipid, fasting blood sugar, and malondialdehyde were measured. At the conclusion of the study, after treatment with FO and SO, total cholesterol, LDL cholesterol (5.6% in FO and 10.8% in SO), and triglyceride levels decreased significantly within each group (*p* < .05). Nonetheless, between‐group differences in systolic BP were significant (*p* < .05). In addition, significant weight loss was observed in both groups. It is suggested that dietary FO may be effective in alleviating some symptoms of MetSyn and lowering blood pressure and lipid peroxidation (Akrami et al., 2018).

Omega‐3 fatty acids are considered essential therapeutic agents and are one of the essential fatty acid varieties. By incorporating cell membranes and preventing autoimmune and inflammatory diseases, they perform a therapeutic role. D‐galactosamine‐sensitized albino rats with hepatitis induced by lipopolysaccharide (LPS) were administered varying doses of flaxseed oil to determine its effect on hepatic inflammation. HPLC was utilized to determine 8‐hydroxyguanosine (8‐OhdG) in the urine. LPS/DGalN significantly increased serum liver functions, MDA, TNF‐, IL‐1, and urinary 8‐OhdG in conjunction with a decrease in liver GSH and SOD compared to the control group, whereas flaxseed oil decreased these parameters. This study concluded that flaxseed oil's high omega‐3 fatty acid content has a significant impact on the prevention of oxidative stress and hepatic inflammation (Hussein et al., 2016).

It has been observed that omega‐3 fatty acids are responsible for reduced hepatic fat accumulation and regulated lipid metabolism. Rezaei et al conducted research to verify this theory; they examined the efficacy of oil of flaxseeds, a rich source of ALA, on liver fat accumulation and potential cardiometabolic disease risk factors in individuals with NAFLD. There were 68 NAFLD patients among the research participants, and a controlled trial experiment was conducted. They were given a hypocaloric (−500 kcal/day) diet and 20 g/day of flaxseed oil in one group and sunflower oil in the other group for a period of 12 weeks. In both groups 1 and 2, the degree of obese liver was diminished (−0.68 for flaxseed oil and −0.22 for sunflower oil). Serum levels of aspartate aminotransferase and alanine aminotransferase were reduced in both groups (*p* < .01). In addition, significantly lower levels of glucose in the blood (*p* = .005) and adipose mass (*p* = .01) were observed in the group given flaxseed oil compared to the group given sunflower oil (*p* = .01). Interleukin‐6 levels did not change significantly in either group, but there was a significant difference between the groups (*p* = .03). Therefore, the analysis concluded that a low‐calorie diet, moderate physical activity, and the use of flaxseed oil may be beneficial for NAFLD patients, as it improves the grade of fatty liver, weight, and interleukin‐6 in comparison to sunflower oil (Rezaei et al., 2020).

## 10 CONCLUSION

The research studies reveal that the components of flaxseeds are beneficial in preventing disease and also play a role in therapeutic uses. This leads to the conclusion that healthy and functional foods should be developed by incorporating flaxseeds in them. A number of various anti‐cancerous outcomes are portrayed in vitro on AML cells by the ENL flaxseed lignan. ENL is a gut metabolite of SDG, it is the main flaxseed lignan having health benefits and protective effects in AML. In addition, additional research is required to discover the health benefits of flaxseed's constituents and to determine the quantity of flaxseed needed to examine its efficacy as a treatment for people, including pregnant and breastfeeding women, as well as to identify any potential side effects of its use in high doses. Some areas are still under exploration to draw valid results but the primary data are encouraging. As there is very few or no evidence of toxicity due to flaxseed supplementation, it supports the argument that it should be included in the daily diet in moderate amounts.

## AUTHOR CONTRIBUTIONS

**Sana Noreen:** Data curation (equal); writing – original draft (equal). **Tabussam Tufail:** Conceptualization (equal); supervision (equal); writing – review and editing (equal). **Huma Bader Ul Ain:** Supervision (equal); validation (equal). **Chinaza Godswill Awuchi:** Project administration (equal).

## FUNDING INFORMATION

No funding was received for this study.

## CONFLICT OF INTEREST STATEMENT

The authors declare no conflict of interest.

## ETHICS STATEMENT

The study does not involve any human or animal testing.

## Data Availability

Data used for this review are available on request through the corresponding author, although all the relevant data have been provided here.
